# Draft Genome Sequence of *Tritrichomonas foetus* Strain K

**DOI:** 10.1128/genomeA.00195-17

**Published:** 2017-04-20

**Authors:** Marlene Benchimol, Luiz G. P. de Almeida, Ana Tereza Vasconcelos, Ivone de Andrade Rosa, Maurício Reis Bogo, Luiza Wilges Kist, Wanderley de Souza

**Affiliations:** a Laboratorio de Ultraestrutura Celular Hertha Meyer, Centro de Ciências da Saúde, Instituto de Biofísica Carlos Chagas Filho, Universidade Federal do Rio de Janeiro, Cidade Universitaria, Rio de Janeiro, Brazil; b Universidade do Grande Rio, UNIGRANRIO, Duque de Caxias, Rio de Janeiro, Brazil; c Laboratório de Bioinformática, Laboratório Nacional de Computação Científica, Petrópolis, Brazil; d Instituto Nacional de Metrologia, Qualidade e Tecnologia, Inmetro, Rio de Janeiro, Brazil; e Laboratório de Biologia Genômica e Molecular, Pontifícia Universidade Católica do Rio Grande do Sul (PUCRS), Porto Alegre, Rio Grande do Sul, Brazil

## Abstract

The protist *Tritrichomonas foetus* (Excavata, Parabasalia) is a parasite that causes bovine and feline trichomonosis. Bovine trichomonosis is a venereal disease that leads to abortion and reproductive problems in herds. Feline trichomonosis affects domestic cats. Here, we report the genome sequence of the *T. foetus* K strain, isolated in Brazil.

## GENOME ANNOUNCEMENT

The protist *Tritrichomonas foetus* (Excavata, Parabasalia) is an important parasite that causes bovine and feline trichomonosis that leads to abortion and other reproductive problems in infected herds, resulting in considerable economic losses. Feline trichomonosis affects domestic cats worldwide. One characteristic feature of this protozoan is the presence of an unusual anaerobic energy-generating organelle, surrounded by two closely apposed membranes, known as hydrogenosomes, among other organelles with unknown functions. All these features make *T. foetus* an excellent model to analyze evolutive aspects of the organization of highly specialized eukaryotic microorganisms. Here, we report the genome sequence of *T. foetus* strain K, isolated in Brazil from an infected bull, which has been the subject of a large number of structural and biochemical studies (1–13). The results obtained are important and allow a comparison with the genome of *Trichomonas vaginalis*, another member of the Trichomonadidae family that causes human trichomonosis and is a highly prevalent sexually transmissible disease.

The parasites were cultivated in Trypticase-yeast extract-maltose (TYM) medium, and the total genomic DNA was purified using the Wizard genomic DNA purification kit (Promega, USA). DNA concentration, purity, and the overall integrity were checked using a spectrophotometer (optical density at 260 nm [OD_260_]/OD_280_ ratio) and by agarose gel electrophoresis. Sequencing was carried out using the Illumina HiSeq platform and 454 GS-FLX Titanium. One shotgun library from 454 and three libraries from Illumina: one paired-end and two mate-pair (3 kb and 8 kb) were prepared. Assembly of the genome was carried out using the AllPaths-LG r47609 software (14) with Illumina reads. Using Illumina and 454 reads, we improved the gap closure with GapFiller 1.11 (15). Using this protocol, a total of 3,730 contigs were generated and assembled into 1,573 scaffolds, totaling 68,472,157 bp. The longest scaffold was 694,095 bp. AllPaths-LG estimated the genome size to be 161,213,455 bp, with 62% repetitive sequences. To improve the assembly, several softwares were used, such as Meraculous, Ray, SOAP*denovo*, and SPAdes. The results were similar to those found using the AllPaths software. The number of repeated sequences makes it impossible to assemble larger contigs.

Automated functional annotation was performed *de novo* using the System for Automated Bacterial Integrated Annotation (SABIA) (16). We identified 7,856 proteins with homology to known proteins from other organisms, as well as 17,497 hypothetical proteins, with a coding sequence (CDS) average length of 1,582 bp. Using KEGG, 72% of the open reading frames (ORFs) were found to be similar to those of *Trichomonas vaginalis*. The results obtained were compared with the genome of *T. vaginalis*, which presents a genome with 65% repetitive sequences (17, 18). In both trichomonads, the superabundance of repeats resulted in a highly fragmented sequence, preventing an investigation of genome architecture (18). The other 28% remaining ORFs have no significant results with any other genome. The assembled genome, together with the functional annotation, is available at http://www.labinfo.lncc.br/index.php/tritrichomonas_foetus (1, 2, 5, 19).

### Accession number(s).

The *Tritrichomonas foetus* genome sequence is available in GenBank. This whole-genome shotgun project has been deposited at DDBJ/ENA/GenBank under the accession MLAK00000000. The version described here is MLAK01000000.
